# Polyphyllin B inhibited STAT3/NCOA4 pathway and restored gut microbiota to ameliorate lung tissue injury in cigarette smoke-induced mice

**DOI:** 10.1186/s12896-024-00837-6

**Published:** 2024-03-08

**Authors:** Qing Wang, Zhiyi He, Jinqi Zhu, Mengyun Hu, Liu Yang, Hongzhong Yang

**Affiliations:** 1 The Affiliated Changsha Central Hospital, Department of Respiratory and Critical Care Medicine, Hengyang Medical School, University of South China, Changsha, Hunan 410004 China; 2 Department of Respiratory and Critical Care Medicine, The First Affiliated Hospital of Guangxi Medical University, Nanning, 530021 Guangxi China

**Keywords:** Polyphyllin B, Cigarette smoke, COPD, Gut microbiota, STAT3/NCOA4 pathway

## Abstract

**Objective:**

Smoking was a major risk factor for chronic obstructive pulmonary disease (COPD). This study plan to explore the mechanism of Polyphyllin B in lung injury induced by cigarette smoke (CSE) in COPD.

**Methods:**

Network pharmacology and molecular docking were applied to analyze the potential binding targets for Polyphyllin B and COPD. Commercial unfiltered CSE and LPS were used to construct BEAS-2B cell injury in vitro and COPD mouse models in vivo, respectively, which were treated with Polyphyllin B or fecal microbiota transplantation (FMT). CCK8, LDH and calcein-AM were used to detect the cell proliferation, LDH level and labile iron pool. Lung histopathology, Fe^3+^ deposition and mitochondrial morphology were observed by hematoxylin–eosin, Prussian blue staining and transmission electron microscope, respectively. ELISA was used to measure inflammation and oxidative stress levels in cells and lung tissues. Immunohistochemistry and immunofluorescence were applied to analyze the 4-HNE, LC3 and Ferritin expression. RT-qPCR was used to detect the expression of FcRn, pIgR, STAT3 and NCOA4. Western blot was used to detect the expression of Ferritin, p-STAT3/STAT3, NCOA4, GPX4, TLR2, TLR4 and P65 proteins. 16S rRNA gene sequencing was applied to detect the gut microbiota.

**Results:**

Polyphyllin B had a good binding affinity with STAT3 protein, which as a target gene in COPD. Polyphyllin B inhibited CS-induced oxidative stress, inflammation, mitochondrial damage, and ferritinophagy in COPD mice. 16S rRNA sequencing and FMT confirmed that *Akkermansia* and *Escherichia_Shigella* might be the potential microbiota for Polyphyllin B and FMT to improve CSE and LPS-induced COPD, which were exhausted by the antibiotics in C + L and C + L + P mice. CSE and LPS induced the decrease of cell viability and the ferritin and LC3 expression, and the increase of NCOA4 and p-STAT3 expression in BEAS-2B cells, which were inhibited by Polyphyllin B. Polyphyllin B promoted ferritin and LC3II/I expression, and inhibited p-STAT3 and NCOA4 expression in CSE + LPS-induced BEAS-2B cells.

**Conclusion:**

Polyphyllin B improved gut microbiota disorder and inhibited STAT3/NCOA4 pathway to ameliorate lung tissue injury in CSE and LPS-induced mice.

**Supplementary Information:**

The online version contains supplementary material available at 10.1186/s12896-024-00837-6.

## Introduction

The global initiative for chronic obstructive pulmonary disease (COPD) was established in the late 1990s to spread awareness of COPD as a major public health problem and to promote its prevention and treatment [1]. Currently, COPD is the fourth leading cause of death in the United States, and smoking is the leading, but not the only, cause of COPD [2, 3]. Despite limited data, patients should continue to use inhaled corticosteroids, long-acting bronchodilators, or chronic macrolides for stable COPD management [4]. Inhaler therapy is the mainstay of COPD treatment, but it should also be complemented by multiple management strategies, including smoking cessation counseling and medication [3]. Therefore, exploring the pharmacologic treatment of cigarette smoke (CSE)-related COPD may help to develop new therapeutic strategies.

The natural history of COPD was complex as a syndrome, which was caused by numerous interacting factors throughout the life cycle, with smoking being the most strongly stimulating feature [5]. Regardless of the Forced expiratory volume in one second (FEV1) trajectory, persistent smoking was strongly associated with disease progression, structural lung disease development, and poor prognosis [5, 6]. During cigarette smoke (CS) exposure, lipid peroxidation and instability iron accumulation were enhanced in the lungs of COPD mice, accompanied by ferritinophagy and non-apoptotic cell death, which is regulated by GPX4 and NCOA4 activity [7]. Ferritinophagy was induced by ferritin degradation and transferrin receptor 1 (TfR1) expression to lead to iron-dependent ferroptosis [8]. Ferritinophagy is crucial for regulating iron homeostasis, but excessive activation of iron autophagy can lead to intracellular iron overload and increase cell sensitivity to iron-induced cell death [9]. The imbalance between oxidants and antioxidants leads to an imbalance in the REDOX environment that seriously affects COPD and its complications [10]. In addition, exposure to diesel exhaust particles resulted in persistent lung inflammation, and microbial alterations in male C57BL/6 mice fed a regular diet (LF) or a high fat (HF) diet in a ROS-RNS-mediated manner, which was exacerbated by concurrent consumption of HF diet [11]. Therefore, regulating the oxidative/antioxidants balance and iron homeostasis related ferritinophagy in CSE-induced mice may be a potential therapy for CSE-related COPD.

The gut-lung axis theory points out that the microecological imbalance of gut microbiota mainly refers to the imbalance of the number and proportion of intestinal microbiota, which is manifested by the increase of intestinal potential pathogenic bacteria and the increase of intestinal endotoxin release and other factors that are involved in the pathogenesis and progression of COPD [12]. In addition, decreased intestinal inflammation, enhanced mitochondrial and ribosomal activity in colonic cells, systematic restoration of abnormal host amino acid metabolism in serum, and suppression of pulmonary inflammation are important ameliorative mechanisms of COPD [13]. Polyphyllin B, a natural compound, is one of the components of the traditional Chinese medicine Chongluo saponin, which plays an anti-tumor role by mediating immunity and inducing apoptosis of cancer cells [14, 15]. Still, the role of Polyphyllin B in COPD-related gut microbial disorder and lung tissue injury remains unknown. Therefore, this study attempted to construct CSE and LPS-induced mice and orally gavage Polyphyllin B to explore its potential mechanism in intestinal microbial disorder and lung tissue injury to supply new insight for the treatment of CSE-related COPD.

## Material and methods

### Network pharmacology analysis

First, the SMILES ID of the Polyphyllin B compound was obtained through the PubChem database (https://pubchem.ncbi.nlm.nih.gov/) [16]. The SMILES ID was imported into the SwissTargetPrediction database (http://www.swisstargetprediction.ch/) [17] and SEA database (http://sea.bkslab.org/) [18] to obtain corresponding compound targets. Then, the PharmMapper database (http://www.lilab-ecust.cn/pharmmapper/) [19] and Genecards database (https://www.genecards.org/) [20] were used to retrieve compound targets for Polyphyllin B. Targets with a possibility score of > 0 from the SwissTargetPrediction database and with a score > 0.5 from the PharmMapper database. After deduplication and correction using the UniProt database (https://www.uniprot.org/) [21], a total of 243 targets were obtained. The "Chronic obstructive pulmonary disease" was used as the keyword to search for human genes in the GeneCards database (https://www.genecards.org/), NCBI database (https://www.ncbi.nlm.nih.gov/) [22], and DisGeNET database (https://www.disgenet.org/) [23]. Among them, target genes from the GeneCards database were selected based on scores to obtain more relevant targets. After merging and removing duplicates from these three databases, a total of 2257 disease-related genes were obtained. The drug targets and disease targets were input into Venny 2.1 software to obtain 124 overlapping targets. These overlapping targets were used as predicted targets for drug action on the disease and subjected to enrichment analysis. These overlapping targets were input into the String database (https://string-db.org/cgi/input.pl) [24] to construct a PPI network, with the biological species set as "Homo sapiens, Confidence score > 0.4". It resulted in a PPI network with 124 nodes, 1074 edges, and an average degree of 17.5. The PPI network was imported into Cystoscape 3.8.0 [25], and topological analysis was performed using the NetworkAnalyzer tool. Based on the degree ranking, genes with scores greater than twice the average score were selected as key targets, resulting in a total of 44 key targets. R 4.0.3 was used to draw a plot for the top 20 targets, with the x-axis representing the degree value of each target. Then, based on the included components, therapeutic diseases, and targets, a compound-disease-target network diagram was constructed using Cytoscape, which helps to understand the complex interactions between components, diseases, and corresponding targets.

### Molecular docking analysis

In recent years, molecular docking has played an important role in obtaining new drug candidates in a short time and at a low cost through computational tools [26]. Based on network pharmacology analysis, the binding affinity between the top 5 ranked gene proteins (ALB, TP53, EGFR, STAT3, ESR1) and Polyphyllin B was predicted using AutoDock VINA 1.1.2 software. Subsequently, visualization analysis was performed using PYMOL and ligplus. The 3D ALB, TP53, EGFR, STAT3 and ESR1 crystal structures were acquired from the protein database (https://www.uniprot.org/). The PyMOL software (2.3.0) was applied to remove the original ligand and water molecules of the target enzymes. Then, the protein was imported into AutoDockTools for hydrogenation, charge calculation and distribution as well as atom type specification. The 3D structure of Polyphyllin B was downloaded from the PubChem (https://pubchem.ncbi.nlm.nih.gov/) in 3D SDF format and imported into ChemBio 3D for further energy-minimization. The ligand molecule was flexible and saved as a PDBQT format to get the most convenient affirmation. AutoDock VINA 1.1.2 software was used to evaluate the binding affinity of Polyphyllin B to proteins. Polyphyllin B could stably bind to the cavity of STAT3 protein and interact with surrounding amino acids (Table 1).

**Table 1:** Molecular docking shows the binding affinity of Polyphyllin B with ALB, TP53, EGFR, STAT3, and ESR1

Polyphyllin B	Binding energy (kcal/mol)	Interacting amino acid residues		
		**hydrogen bonds**	**hydrophobic interactions**	**Van der Waals forces**
ALB	-10.7	A chain: GLN 390, GLU 393, GLN 397 B chain: ASP 375 ALA 371 VAL 310	A chain: LYS 389 B chain: PHE 374 ALA 306 HIS 338 ARG 337 LEU 302 MET 298	A chain: ASN 386 B chain: VAL 373 VAL 310 LYS 372 HIS 367 GLU 311 LEU 305 SER 304 ASP 301 ALA 300
TP53	-9.1	A chain: SER 166 GLN 100 B chain: ARG 196 GLU 198	B chain: LEU 137 TYR 239 HIS 178 HIS 179 ALA 138	A chain: MET 169 THR 102 LEU 252 LYS 164 LYS 101 B chain: ASN 235 ASP 184 ASP 186 THR 140 MET 237 GLY 199 MET 237 LYS 139
EGFR	-9.5	A chain: ASP 800 MET 793 GLY 719	A chain: ARG 841 LEU 718	A chain: ASN 842 LEU 844 ALA 743 LEU 792 LEU 1001 GLY 796 LYS 745 PHE 997 VAL 726 SER 720 GLY 721 LYS 875 THR 725 PRO 794 TRP 880 ASP 916 GLY 917 LYS 879 LYS 913
STAT3	-11.2	A chain: TYR 446 GLU 357 HIS 447	A chain: LYS 282 B chain: LEU 358 LYS 363	A chain: GLU 286 GLN 289 GLY 449 GLN 448 LEU 358 B chain: VAL 393 TYR 446 TYR 360 GLN 361 GLN 448 GLY 449 TYR 360 GLU 357
ESR1	-8.6	A chain: LYS 362 PHE 367 LEU 308 LEU 310 ASP 369	A chain: HIS 550 ALA 551 ARG 548 ALA 546 VAL 368 ARG 363 ALA 318	HIS 547 ALA 322 VAL 364 SER 309 ALA 307 GLY 366 PRO 365 GLN 314 HIS 474 ALA 322

The protein structure information was shown as follow. ALB: PDB ID: 6HSC (https://www.rcsb.org/structure/6HSC). TP53: PDB ID: 5AOK (https://www.rcsb.org/structure/5AOK). EGFR: PDB ID:3QWQ (https://www.rcsb.org/structure/3QWQ). STAT3: PDB ID: 6TLC (https://www.rcsb.org/structure/6TLC). ESR1: PDB ID: 5FQP (https://www.rcsb.org/structure/5FQP). And the information about grid box was showed in the following. ALB: center_x = -58.861, center_y = -1.95, center_z = 43.619, size_x = 47.25, size_y = 47.25, size_z = 47.25. The spacing between each grid point is 0.375 Å. TP53: center_x = 114.517, center_y = 87.056, center_z = -29.785, size_x = 47.25, size_y = 47.25, size_z = 47.25. The spacing between each grid point is 0.375 Å. EGFR: center_x = 19.337, center_y = 26.345, center_z = 14.608, size_x = 47.25, size_y = 47.25, size_z = 47.25. The spacing between each grid point is 0.375 Å. STAT3: center_x = 0.265, center_y = 32.908, center_z = 33.475, size_x = 47.25, size_y = 47.25, size_z = 47.25. The spacing between each grid point is 0.375 Å. ESR1: center_x = 22.783, center_y = 5.383, center_z = 22.087, size_x = 47.25, size_y = 47.25, size_z = 47.25. The spacing between each grid point is 0.375 Å.

### Establishment and treatment of CSE + LPS mice model [27]

The commercial unfiltered cigarettes (Xiangsiyan from Hunan Tobacco Industry Co., LTD., China) used in this study contained 11 mg of tar and 0.9 mg of nicotine per cigarette. In brief, C57BL/6 J mice (male, 8-week-old), purchased from Hunan SJA Laboratory Animal Co., Ltd, were divided into the following groups: control (saline), CSE + LPS group (C + L, CSE and LPS treatment), CSE + LPS + PB group (C + L + PB, CSE, LPS and PB treatment), 6 mice/group (Supplementary Fig. 1). The CSE + LPS model was constructed as follows: Mice were placed in a perspex chamber (50 × 60 × 70 cm), which was covered with disposable filters. During the first week, the mice received four cigarettes daily (8:00, 12:00, 16:00, and 20:00 each day) five days a week. Then, mice were exposed to smoke from six cigarettes (8:00, 10:30, 13:00, 15:30, 18:00 and 20:30 each day) a day (five days a week) until week 6. At the end of weeks 3 and 5, mice were injected intratracheally with 750 ng/kg LPS (LPS dissolved in 50 μL saline, Sigma-Aldrich) or 50 μL saline. The mice in the C + L + PB group were treated with 150 mg/kg/d Polyphyllin B (CAS No.50773–42-7, CS-N2389, Chemstan) by oral gavage. At the end of the 7th week, the mice were euthanized by intraperitoneal injection of 150 mg/kg sodium pentobarbital to collect blood, bronchoalveolar lavage fluid (BALF), feces, and lung tissue.

### Fecal microbiota transplantation (FMT) [28]

Mice were randomly divided into CSE + LPS (C + L) group, FMT (C + L + P, feces from C + L + PB mice was transplanted to C + L group mice) group, ABX (C + L + A, antibiotic treatment to remove intestinal microorganisms as control) group, ABX + FMT (C + L + A + P, feces from C + L + PB group was transplanted to C + L + A group) group, 6 mice/group (Supplementary Fig. 1). Antibiotics broad-spectrum confine [vancomycin (0.5 g/liter), neomycin sulfate (1 g/liter), metronidazole (1 g/liter), and ampicillin (1 g/liter)] were used to remove gut microbes for 5 days and no treatment for 2 days in the C + L + A and C + L + A + P groups, and then COPD models were developed. Mice in the C + L + P and C + L + A + P groups were irrigated with feces from mice in the C + L + PB group to observe the activity of mice and monitor the test indicators. For FMT, 200 mg of fresh feces were collected from mice in the C + L + PB group and re-suspended in 5 mL PBS. The homogenate was passed through a 40 µm aperture nylon filter to remove large particles and fibrous material. The suspension was centrifuged for 2 min, and 200 μL of resuspended feces was immediately transplanted into recipient mice using oral tube feeding twice a week [29]. All animal experiments complied with ARRIVE guidelines and the samples used in this research were approved by the Ethics Committee of the University of South China (2023–105).

### Cell experiments and grouping

BEAS-2B cells (AW-CNH004, abiowell) were cultured in DMEM containing 10% fetal bovine serum (FBS) + 1% penicillin–streptomycin (P/S) solution at 37℃, 5% CO_2_ and saturated humidity. P/S solution was designed to inhibit bacterial growth and avoid cell contamination. In both the presence and absence of standard antibiotics, cell experiments should be conducted with caution inside a laminar flow hood. The operating environment also was subjected to ultraviolet sterilization, and all instruments and consumables underwent high-pressure sterilization. BEAS-2B cells were randomly divided into Control group (normal culture), cigarette smoke (CSE) group (5% CSE extract, 48 h), CSE + PB group (1.25 μM PB + 5% CSE extract, 48 h). CSE + LPS group (1 µg/ml LPS + 5% CSE extract, 48 h), CSE + LPS + PB group (1.25 μM PB + 1 µg/ml LPS + 5% CSE, 48 h) (Supplementary Fig. 1). The preparation process of CSE extract is as follows: A cigarette was connected to a 60 mL syringe through a rubber tube and lit, and three tubes of thick smoke in the second half of the cigarette were extracted. The extracted cigarette smoke was passed into the prepared vacuum container containing 5 mL DMEM culture fluid, and the container was gently stirred to dissolve the smoke in the culture fluid fully. The resulting suspension was filtered through a 0.22 µm filter and used as the CSE extract solution (100% CSE extract). The 5% CSE was used to treat BEAS-2B cells as the previous report [27].

To investigate the role of the STAT3/NCOA4 pathway in the CSE + LPS-induced BEAS-2B cell model, the cells were randomly divided into the following groups: CSE + LPS, CSE + LPS + PB, CSE + LPS + PB + oe-NC, and CSE + LPS + PB + oe-STAT3 (Supplementary Fig. 1). The cells in the CSE + LPS and CSE + LPS + PB groups were treated as mentioned above. In addition, for the cells in the CSE + LPS + PB + oe-NC and CSE + LPS + PB + oe-STAT3 groups, lipo 2000 was used for oe-NC and oe-STAT3 transfection, respectively, alongside the aforementioned treatment. The cells in each group were treated for a total of 48 h. Subsequently, cell samples were collected for further analysis.

### Cell counting kit-8 (CCK8)

The cells were seeded in 96-well plates at a density of 5 × 10^3^ cells/well, 100 μL per well. CCK8 (NU679, Japanese colleague) was added to each well at 10 μL/well. After further incubation at 37℃ and 5% CO_2_ for 4 h, the absorbance value (450 nm) was detected by the Bio-Tek microplate reader (MB-530, HEALES). Optical density represents the vitality of cells.

### Lactate dehydrogenase (LDH) assay

The culture flask filled with cells was placed on ice, and the culture fluid was sucked out with a pipette. The level of LDH release in the culture fluid was detected by a colorimetric method according to the LDH (A020-1, Nanjing Jiangcheng Bioengineering Institute) kit instruction [30].

### Calcein-AM assay

Each group of cells was digested with trypsin without EDTA to collect 1 × 10^6^ cells. The basal medium was prepared with 0.2 μM Calcein-AM (17,783-1MG, Sigma-Aldrich) working solution. The cell precipitation was resuspended with 1 mL of working solution for incubation. Cell precipitation was obtained by centrifugation, washed twice with PBS and incubated with trypan blue (1,117,320,025, Sigma-Aldrich). The Bio-Tek microplate reader (MB-530, HEALES) was used to measure the baseline fluorescence signal at 488 nm excitation and 517 nm emission. The fluorescence was measured by adding 100 μM 2, 2-bipyridine (D216305, Sigma-Aldrich).

### Hematoxylin–eosin (HE) and Prussian blue staining

Lung tissue was fixed and sectioned to 5 μm sections. Sections were baked at 60℃ for 12 h. Sections were dewaxed to water and stained with hematoxylin (abiowell) and eosin (abiowell). In addition, Prussian blue kit (abiowell) was applied to incubate and stain at 37℃ for 25 ~ 30 min, followed by nuclear solid red dye for 5 ~ 10 min. Then, it was rinsed with distilled water. The sections were dehydrated with gradient alcohol (95 ~ 100%) and sealed by neutral balsam. The sections were examined on a microscope (BA210T, Motic).

### Immunohistochemistry (IHC)

Lung tissue sections were immersed in 0.01 M citrate buffer (pH 6.0) and heated in a microwave for 20 min. Subsequently, they were allowed to cool to room temperature. Endogenous enzymes in sections were inactivated with 1% periodate acid. Appropriate dilutions of anti-4-HNE (1:100, ab46545, Abcam, UK) were added to sections at 4℃ overnight. 50–100 μL IgG antibody was added to sections for incubation at 37℃. DAB working solution (Zhongshan Jinqiao) was added to sections for color development. After counterstaining with hematoxylin (abiowell), the sections were sealed by neutral balsam and observed by microscope (BA410T, Motic).

### Immunofluorescence (IF)

After cell climbing sheets, it was fixed with 4% paraformaldehyde for 30 min. Cell climbing sheets were blocked with 5% BSA. Appropriate dilutions of anti-LC3 (1:100, 14,600–1 AP, proteintech, USA) and anti-Ferritin (1:50, ab11017, Abcam, UK) were added to cell climbing sheets for incubation. Then, 50 ~ 100 μL of anti-Goat IgG (1:100, SA00003-3, proteintech, USA) and anti-Rabbit IgG (1:200, SA00013-8, proteintech, USA) were added to cell climbing sheets for incubation. The sheets were stained with DAPI working solution (abiowell) and sealed with buffer glycerin (abiowell) to observe under a microscope (BA410T, Motic).

### Transmission electron microscope (TEM)

Tissues were fixed in glutaraldehyde (2.5%) and osmic acid (1%, 18,456, TED PELLA INC) for 6–12 h and 1–2 h, respectively. The tissues were dehydrated with gradient ethanol (30 ~ 100%) and propylene oxide (M25514, Shanghai Myrell Chemical Company). Subsequently, the tissues were immersed in propylene oxide: epoxy resin (1:1) and pure epoxy resin for 1–2 h and 2–3 h for embedding and oven baked for 60 h. The embedded block was taken out and repaired. Then, ultrathin sections were cut. The copper mesh was retrieved. The sections were stained for the electron (lead and uranium). Finally, sections were observed with a transmission electron microscope (7700, Hitachi). Images were recorded with a digital camera (ER-B, AMT).

### ELISA

Blood and BALF specimens should be placed at room temperature for 2 h and centrifuged at 1000 g for 15 min at 2–8℃ to collect the supernatant. Lung tissue (100 mg) samples were washed with 1 × PBS to remove blood stains. The tissue was cut and put into the tissue grinder to make homogenization with 1 mL of 1 × PBS and then placed at -20℃ overnight. After repeated freezing and thawing treatment twice to destroy the cell membrane, the tissue homogenate was centrifuged at 5000 g for 5 min at 2–8℃ to take the supernatant for detection. TNF-α (KE10002, Proteintech), IL-6 (KE10007, Proteintech), IFN-γ (KE10001, Proteintech), IgA (88–50,450, Thermo Fisher Scientific), IgG (88–50,400, Thermo Fisher Scientific), LPS (MOEB2540, Assay Genie), ROS (E004-1–1, NJJCBio) and nitrotyrosine (NT, RJ17730, RENJIEBIO) kits were used to analyze the levels of TNF-α, IL-6, IFN-γ, IgA, IgG, LPS, ROS and NT on the Bio-Tek microplate reader (MB-530, Huisong).

### RT-qPCR

At the end of the experiment, lung tissues were collected, and total RNA was extracted by TRIzol reagent (Thermo, USA). The cDNA was synthesized using a Kit (HiFiScript cDNA Synthesis Kit, CoWin Biosciences, China). The primer sequence was as follows: FcRn (173 bp): F-TATTAAATGGTCAGAAGAGGGGG, R-GATTTCCGTCTCAGGCCACT; pIgR (154 bp): F-AGAACTCCAGGTTGCCGAAG, R-CTTGTTGCTCCACTTGCACC; STAT3 (109 bp): F- CAATACCATTGACCTGCCGAT, R-GAGCGACTCAAACTGCCCT; NCOA4 (165 bp): F- CTAAGGTCCGCTCGGATCAC, R-GCCCGAAGTACTCCACCAAT, and GAPDH (122 bp): F-GCGACTTCAACAGCAACTCCC, R-CACCCTGTTGCTGTAGCCGTA. The relative mRNA expression levels of FcRn, pIgR, STAT3 and NCOA4 were analyzed by UltraSYBR Mixture (CW2601, Beijing, China) and 2^−ΔΔCT^ method.

### Western blot

Lung tissues of mice and cells in different treatment groups were collected, and the concentration of protein was determined by radioimmunoprecipitation analysis (RIPA, AWB0136, abiowell, China) and the "BCA" method. Protein was separated by 12% SDS-PAGE. Then, it was transferred to a polyvinylidene difluoride membrane, which was activated with methanol, and blocked with 5% nonfat milk (AWB0004, abiowell, China) for at least 1 h at room temperature. The membranes were then incubated overnight at 4℃ with the primary antibody. The primary antibody consisted of anti-p-STAT3 (1:1000, ab267373, Abcam, UK), anti-STAT3 (1:1000, ab109085, Abcam, UK), anti-TP53 (1:10,000, 60,283–2-Ig, proteintech, USA), anti-EGFR (1:20,000, 66,455–1-Ig, proteintech, USA), anti-ESR1 (1:1000, 21,244–1-Ap, proteintech, USA), anti-Ferritin (1:4000, 10,727–1-AP, proteintech, USA), anti-NCOA4 (1:3000, ab86707, Abcam, UK), anti-GPX4 (1:2000, 67,763–1-Ig, proteintech, USA), anti-TLR2 (1:1000, 66,645–1-Ig, proteintech, USA), anti-TLR4 (1:1000, 19,811–1-AP, proteintech, USA), anti-P65 (1:2000, 66,535–1-Ig, proteintech, USA), and anti-β-actin (1:5000, 66,009–1-Ig, Proteintech, USA). Then the membrane was incubated with secondary anti-Mouse IgG (1:5000, AWS0001, Abiowell, China) and anti-Rabbit IgG (1:5000, AWS0002, Abiowell, China) at 37℃ for 90 min. Then, SuperECL Plus (AWB0005, abiowell, China) was used for visualization and imaging analysis. Full-length blots are presented in Supplementary Fig. 2.

### 16S rRNA sequence

DNA in fecal samples was extracted by a DNA extraction kit (CAT. #DP328-02, TIANGEN.). 16S rRNA gene primers (V3-V4 region, 357F 5'- ACTCCTACGGRAGGCAGCAG-3' and 806R 5'-GGACTACHVGGGTWTCTCATAT-3') and phusion enzyme (K1031, APExBIO) were applied to PCR amplification, adding adaptor and library construction. Illumina novaseq6000 PE250 was used for mixed sequencing to obtain Raw Data. Qiime 2 (2020.2) software was used to call DADA2 for Raw Data quality control to obtain a valid sequence (Clean Data). The alpha diversity and beta diversity (PCA, Anosim) index of each sample was calculated using Qiime 2 software. Species were annotated for each ASV/OTU sequence referencing the silva-132–99 database. The jvenn (http://www.bioinformatics.com.cn/static/others/jvenn/example.html) page and R software (VennDiagram package) were used for samples or between groups of common and unique ASVs visualization. LDA Effect Size (LefSe, https://github.com/SegataLab/lefse) was used to analyze microbiota differences between groups.

### Data statistics and analysis

SPSS 21.0 (IBM, USA) was used for the statistical analysis of the data in this study. Measurement data were presented as mean ± standard deviation. Normality and homogeneity of variance tests were performed. Unpaired t-test was used for comparison between groups. One-way analysis of variance or analysis of variance for repeated measures was used for comparison between groups. Tukey's post-test was used for comparison. *P* < 0.05 indicated statistical significance.

## Results

### Network pharmacology and molecular docking analysis of the potential interactive targets between Polyphyllin B and COPD

**Fig. 1:**
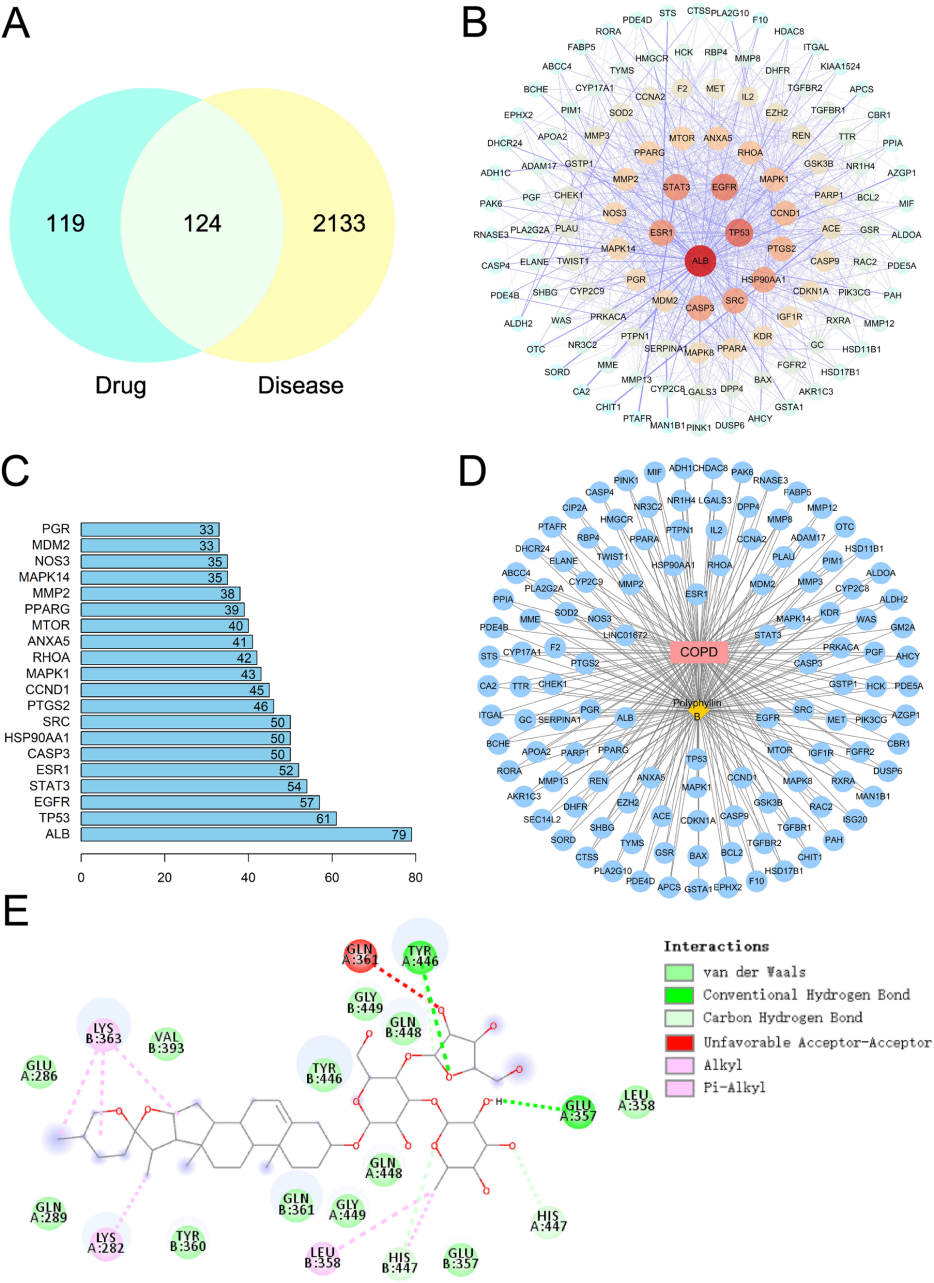
Network pharmacology and molecular docking analysis of the interaction and binding between Polyphyllin B and COPD target genes. **A** Venn diagram showing the number of shared target genes between Polyphyllin B and COPD. **B** PPI network construction of shared target genes. **C** Degree of target genes. **D** Interaction network between Polyphyllin B and COPD target genes. **E** Binding between Polyphyllin B and STAT3 protein

Based on network pharmacology analysis, it was found that Polyphyllin B shares 124 target genes with COPD disease (Fig. 1A). The 124 shared target genes were input into the String database to obtain a PPI network (Fig. 1B). NetworkAnalyzer topology analysis and degree sorting based on the PPI network identified 44 key target genes (Fig. 1C). Among them, ALB, TP53, EGFR, STAT3, and ESR1 were the top 5 key target genes (Fig. 1B-C). Additionally, the Polyphyllin B, target gene, and COPD disease interaction network diagram was constructed using Cytoscape 3.8.0 (Fig. 1D). Molecular docking analysis showed that Polyphyllin B had a good binding affinity with STAT3 protein out of the top 5 key target genes (Table 1, Fig. 1E). Based on this, we speculate that Polyphyllin B might regulate the progression of COPD by targeting STAT3.

### Polyphyllin B improved inflammation in CSE and LPS-induced mice

**Fig. 2:**
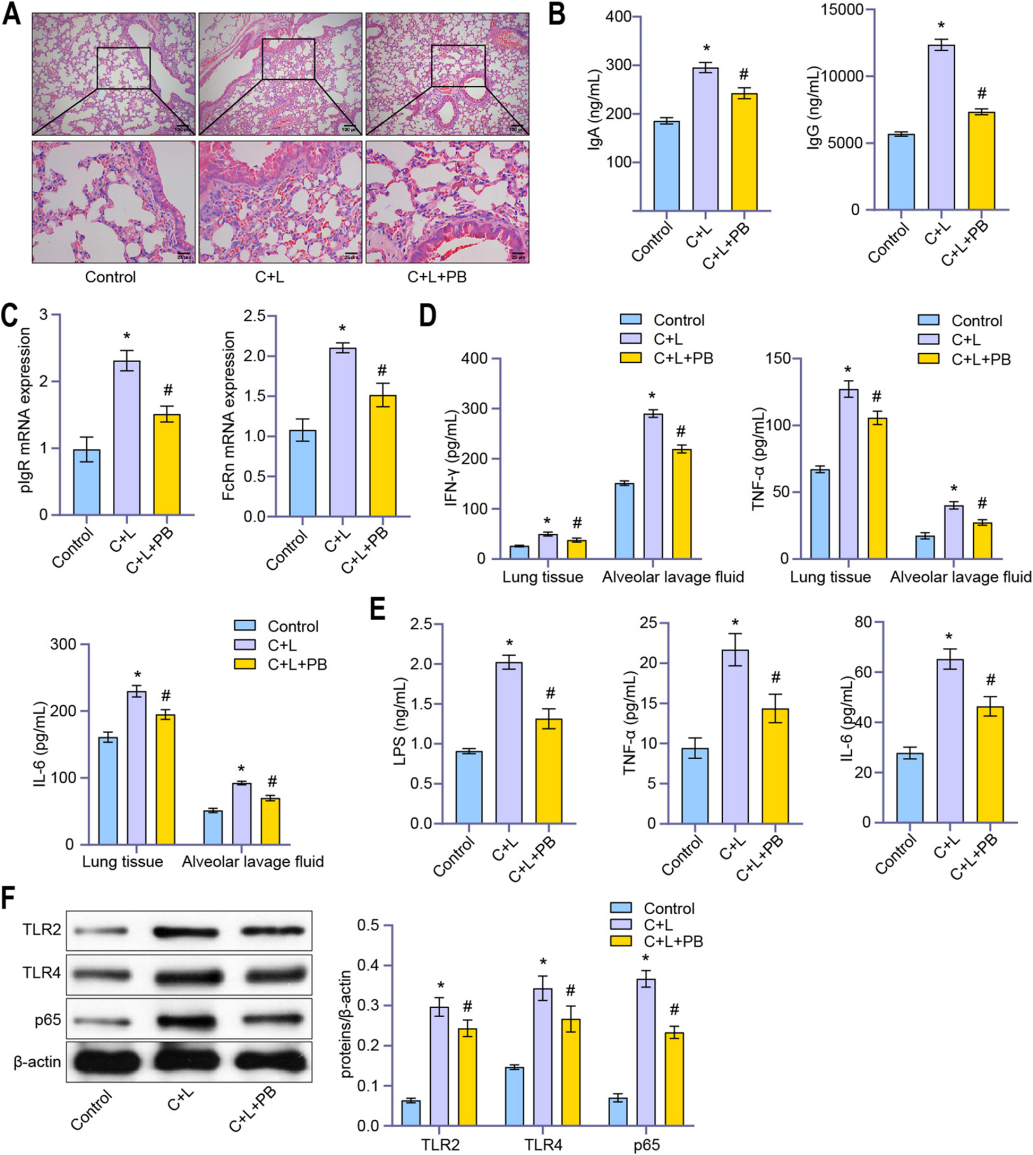
Polyphyllin B inhibited lung and peripheral blood inflammation in CSE and LPS-induced mice. **A** HE staining was applied to observe the pathology of lung tissue. **B** The levels of IgA and IgG in BALF were analyzed by ELISA. **C** RT-qPCR was applied to detect the expression of pIgR and FcRn in mouse lung tissues. **D** The levels of IFN-γ, TNF-α and IL-6 in BALF and lung tissue homogenate were analyzed by ELISA. **E** ELISA was applied to detect the LPS, TNF-α and IL-6 levels in peripheral blood. **F** The expressions of TLR2, TLR4 and p65 in lung tissue were detected by western blot. Full-length blots/gels are presented in Supplementary Fig. 2A. *P < 0.05 vs Control, #*P* < 0.05 vs C + L. *N* = 6 mice/group

HE staining showed that the lung tissue of C + L mice was overinflated, with multiple bullae of different sizes on the surface, the alveolar wall was thinner, and the alveolar cavity was enlarged (Fig. 2A). Polyphyllin B treatment improved lung histopathology in C + L mice (Fig. 2A). In addition, we found CSE and LPS-induced increases in IgA and IgG levels in the alveolar lavage fluid of mice, as well as increased expression of pIgR and FcRn in tissues (Fig. 2B, C). Polyphyllin B treatment inhibited the secretion of IgA and IgG, as well as the expression of pIgR and FcRn in C + L mice (Fig. 2B, C). Polyphyllin B inhibited the CSE and LPS-induced increase of IFN-γ, IL-6 and TNF-α levels in BALF and lung tissue homogenate, and the increase of LPS, TNF-α and IL-6 levels in peripheral blood of mice (Fig. 2D, E). The expression of TLR2, TLR4 and p65 protein was induced by CSE and LPS, which was reversed by Polyphyllin B treatment (Fig. 2F). These results demonstrated that Polyphyllin B inhibited lung and peripheral blood inflammation in CSE and LPS-induced mice.

### Polyphyllin B regulated oxidative stress, mitochondrial damage and ferritinophagy in CSE and LPS-induced mice

**Fig. 3:**
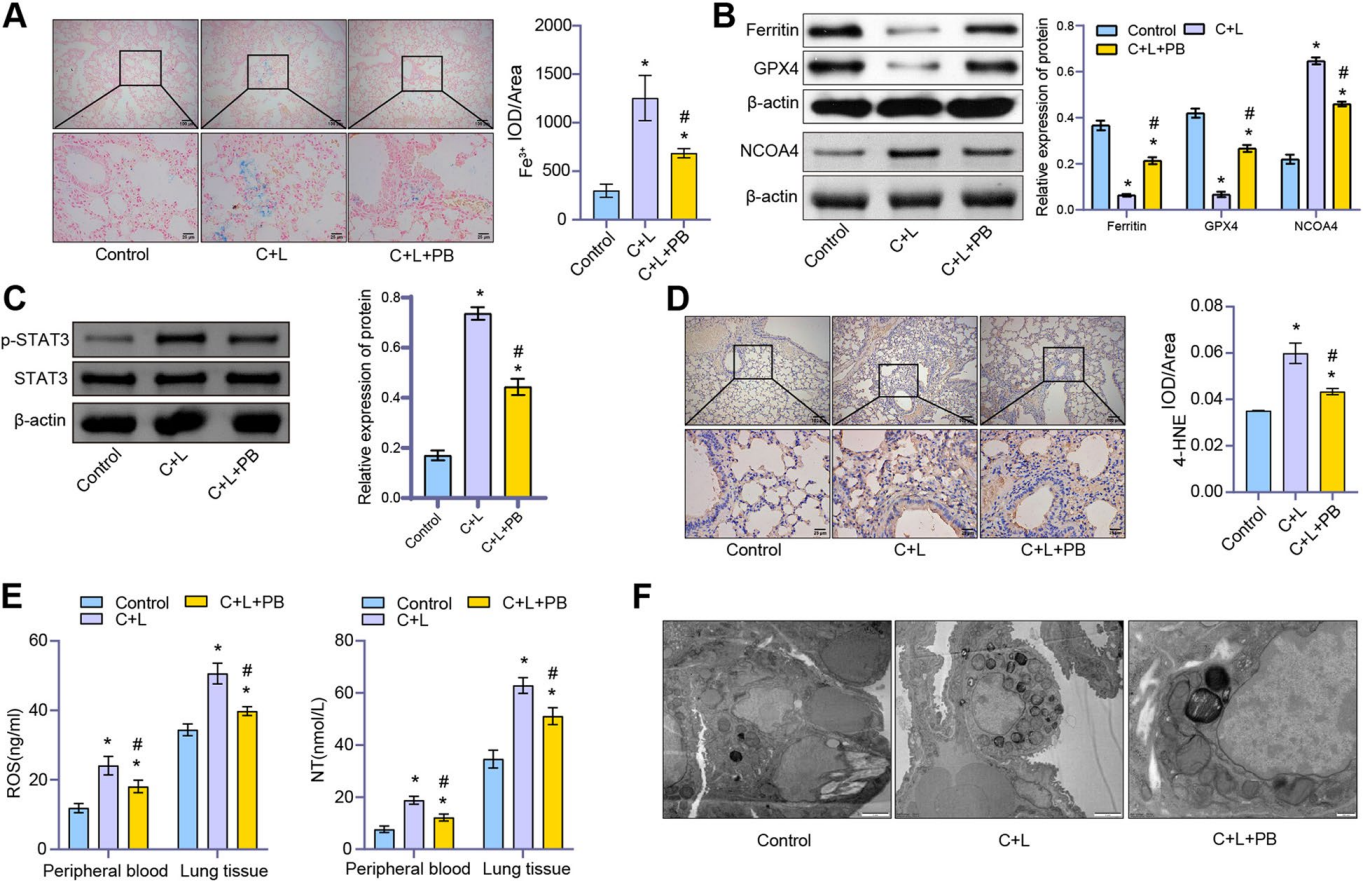
Polyphyllin B regulated CSE and LPS-induced oxidative stress, mitochondrial damage and ferritinophagy in mice. **A** Prussian blue staining. **B** and **C** The expressions of ferritin, NCOA4, p-STAT3, and GPX4 in lung tissue were detected by western blot. Full-length blots/gels are presented in Supplementary Fig. 2B and C. **D** The expression of 4-HNE in lung tissue was detected by immunohistochemistry. **E** The levels of ROS and nitrotyrosine in peripheral blood and lung tissue were detected by ELISA. **F** TEM images showed the changes of mitochondria in lung tissues. *P < 0.05 vs Control, #*P* < 0.05 vs C + L. *N* = 6 mice/group

Prussian blue staining showed obvious deposition of Fe^3+^ in lung tissue, and Polyphyllin B treatment inhibited the deposition of Fe^3+^ in lung tissue of CSE and LPS-induced mice (Fig. 3A). The expression of ferritin and GPX4 was decreased, and the expression of NCOA4 and p-STAT3 was increased in lung tissues of C + L mice (Figs. 3B and C). In addition, Polyphyllin B promoted the expression of ferritin and GPX4, and inhibited the expression of NCOA4 and p-STAT3 in lung tissues (Figs. 3B and C). Immunohistochemical analysis showed that CSE and LPS increased 4-HNE expression in mouse lung tissue, and Polyphyllin B administration significantly inhibited the expression of 4-HNE (Fig. 3D). At the same time, we found that CSE and LPS induced an increase in the levels of ROS and NT in peripheral blood and lung tissue of mice, and administration of Polyphyllin B decreased the levels of ROS and nitrotyrosine in peripheral blood and lung tissue of C + L mice (Fig. 3E). CSE and LPS induced mitochondrial damage in lung tissue of mice, which was inhibited by the administration of Polyphyllin B (Fig. 3F). These results demonstrated that Polyphyllin B inhibited oxidative stress and mitochondrial damage, and promoted ferritinophagy in CSE and LPS-induced mice.

### Polyphyllin B affected the number of gut microbiota in CSE and LPS-induced mice

**Fig. 4:**
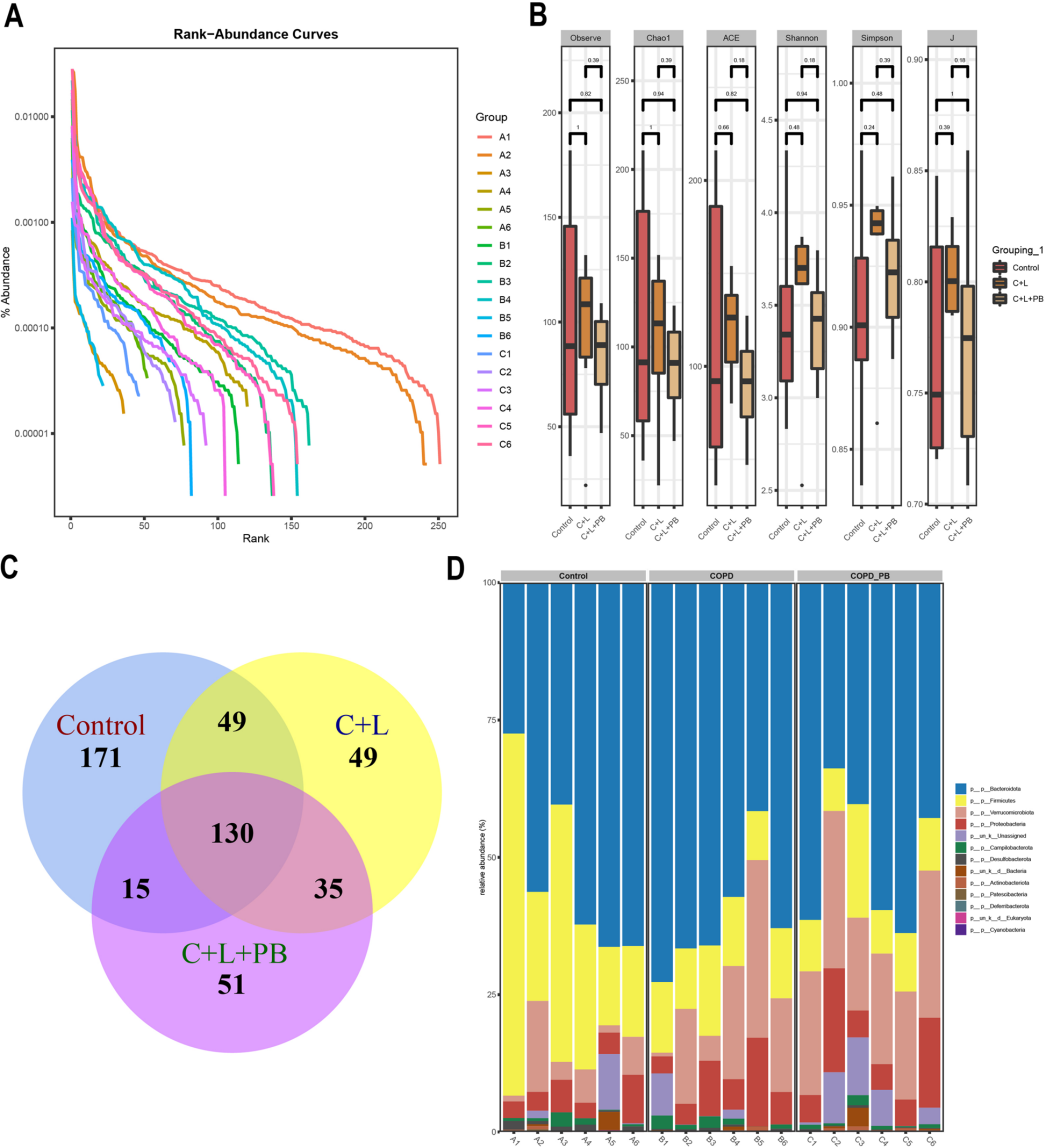
Polyphyllin B affected the number of intestinal microbiota in CSE and LPS-induced mice. **A** Rank abundance curve assesses sequencing depth. **B** Alpha diversity index. **C** Venn diagram shows changes in microbial population. **D** Microbiota abundance at phylum level. *N* = 6 mice/group

The abundance percentage gradually decreased with the increase of sequencing depth (Fig. 4A). Alpha diversity index analysis showed that microbial diversity changed, but there was no significant difference (Fig. 4B). The microbial quantity statistics showed that the intestinal microbial quantity of C + L mice decreased, and Polyphyllin B administration reduced the intestinal microbial quantity of C + L mice (Fig. 4C). At the phylum level, we found Bacteroidota, Firmicutes, Verrucomicrobiota, Proteobacteria, Campilobacterota, Desulfobacterota, Actinobacteriota, Patescibacteria, Deferribacterota and Cyanobacteria were enriched (Fig. 4D). These results demonstrated that Polyphyllin B reduced the number of intestinal microbiota in CSE and LPS-induced mice.

### Polyphyllin B affected the abundance of gut microbiota in CSE and LPS-induced mice

**Fig. 5:**
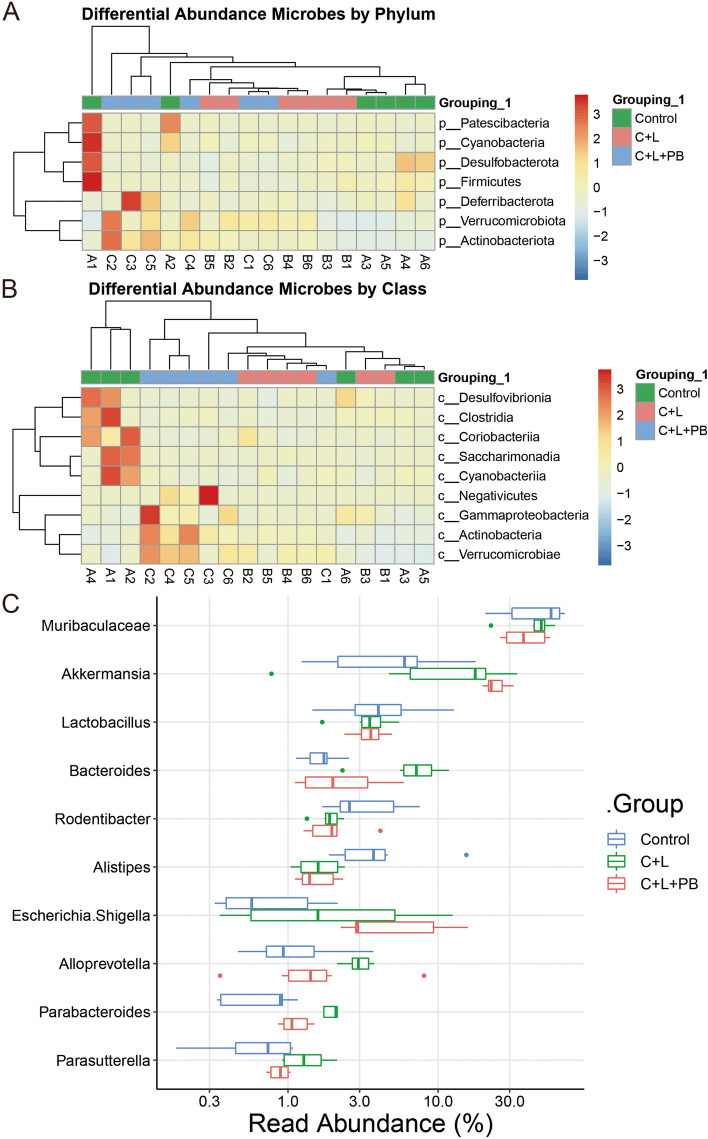
Polyphyllin B affected the abundance of gut microbiota in CSE and LPS-induced mice. **A**-**B** The Heatmap shows the variation of flora abundance at phylum and class levels. **C** The changes in abundance of microbiota at genus level. *N* = 6 mice/group

The Patescibacteria and Cyanobacteria abundance decreased in the C + L group compared to Control group (Fig. 5A). Deferribacterota abundance was up-regulated in the C + L + PB group compared to the C + L group (Fig. 5A). At the class level, the Saccharimonadia, Cyanobacteriia, Clostridia, and Desulfovibrionia abundance decreased significantly in the C + L group compared with Control group (Fig. 5B). Compared with C + L group, Negativicutes was significantly up-regulated in C + L + PB group, while Alphaproteobacteria was significantly down-regulated in C + L + PB group (Fig. 5B). At the genus level, the abundances of *Muribaculaceae*, *Rodentibacter* and *Alistipes* were significantly decreased, the abundance of *Akkermansia*, *Bacteroides*, *Escherichia.Shigella*, *Alloprevotella*, *Parabacteroides* and *Parasutterella* were significantly increased in the C + L group compared with the Control group (Fig. 5C). Compared with C + L group, the *Akkermansia* and *Escherichia.Shigella* were significantly up-regulated, the *Muribaculaceae*, *Bacteroides*, *Alloprevotella*, *Parabacteroides*, and *Parasutterella* were significantly down-regulated in C + L + PB group (Fig. 5C). These studies proved that Polyphyllin B improved the abundance of gut microbiota in CSE and LPS-induced mice.

### FMT proved that Polyphyllin B regulated gut microbiota to inhibit ferritinophagy and improve lung injury in CSE amd LPS-induced mice

**Fig. 6:**
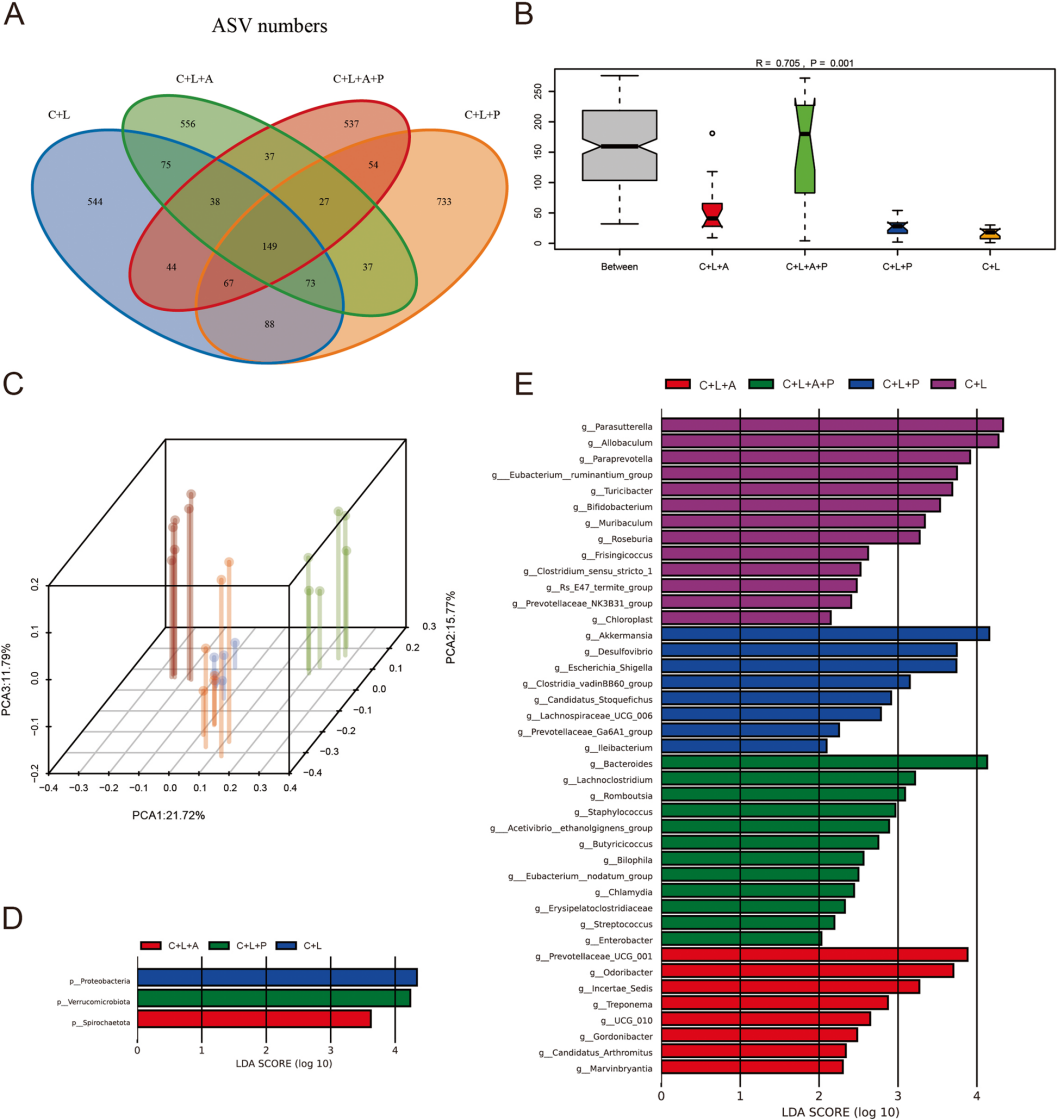
FMT changed the quantity and abundance of gut microbiota in CSE and LPS-induced mice. **A** Venn diagram shows the change of microbial quantity. **B** Anosim analysis. **C** PCA analysis. (D-E) Lefse analysis of different microorganisms at phyla level (**D**) and genus level (**E**). *N* = 6 mice/group

**Fig. 7:**
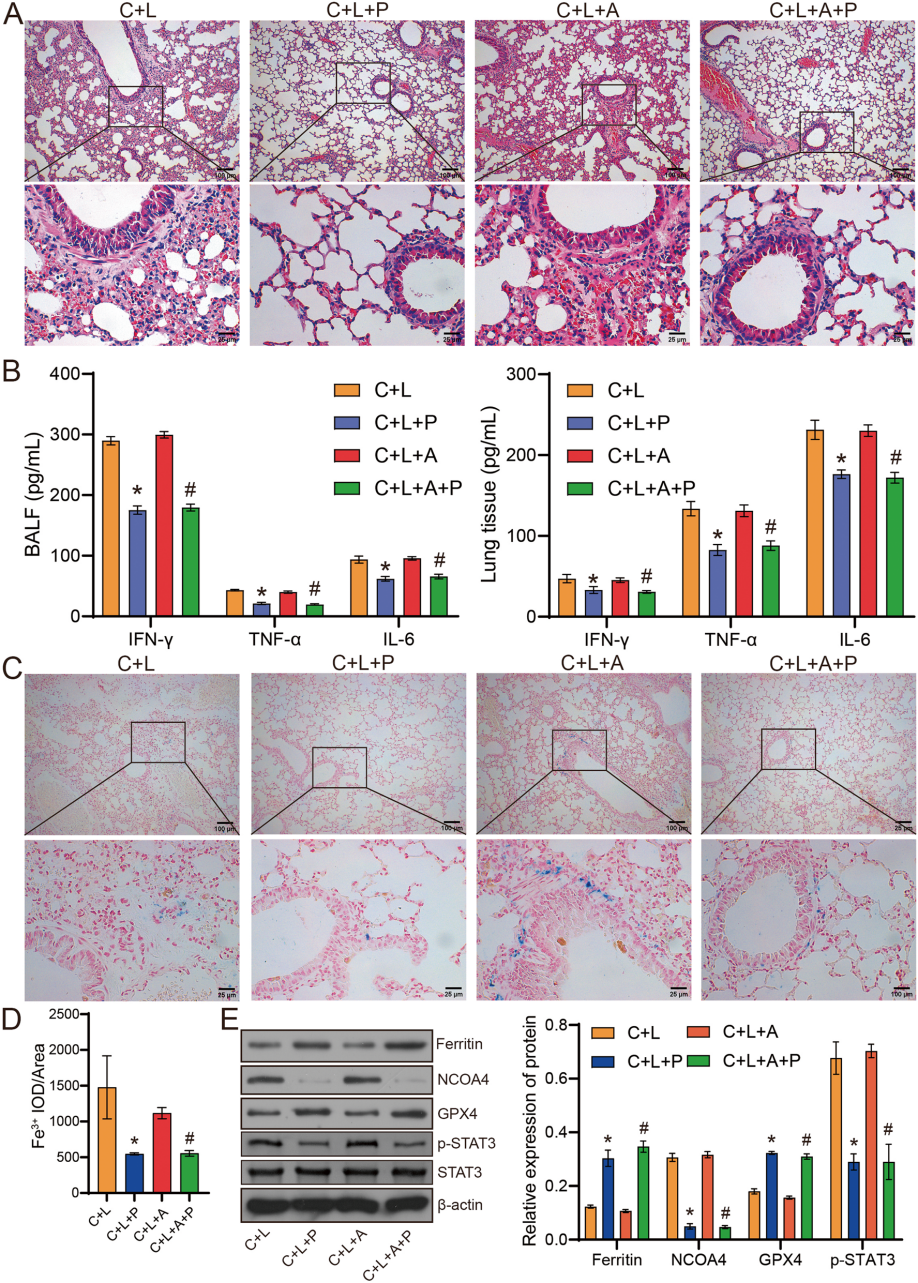
FMT improved gut microbiota disorder and lung injury in CSE and LPS-induced mice. **A** HE staining was used to observe the pathological changes of lung tissue. **B** The levels of IFN-γ, TNF-α and IL-6 in BALF and lung tissue homogenate were detected by ELISA. **C** and **D** Prussian blue staining. **E** The ferritin, NCOA4, p-STAT3, and GPX4 expression in mouse lung tissue were detected by western blot. Full-length blots/gels are presented in Supplementary Fig. 2D. *P < 0.05 vs C + L, #*P* < 0.05 vs C + L + A. *N* = 6 mice/group

The microbial quantity statistics showed that compared with the C + L group, the intestinal microbial quantity of mice in C + L + A group was decreased, and the intestinal microbial quantity of mice in C + L + P group was increased (Fig. 6A). Compared with C + L + A group, the number of intestinal microorganisms in C + L + A + P group decreased slightly (Fig. 6A). Anosim and PCA analysis showed significant differences between groups, and high similarity of samples within groups (Fig. 6B, C). lefse analysis showed that Proteobacteria was significantly enriched in C + L group, Verrucomicrobiota was enriched in C + L + P group, and Spirochaetota was significantly enriched in C + L + A group (Fig. 6D). At genus level, the abundance of *Akkermansia*, *Desulfovibrio*, *Escherichia_Shigella*, *Clostridia vadinBB60 group*, *Candidatus Stoquefichus*, *Lachnospiraceae UCG 006*, *Prevotellaceae Ga6A1 group* and *Ileibacterium* were enriched in the C + L + P group (Fig. 6E). The abundance of *Prevotellaceae UCG 001*, *Odoribacter*, *Incertae Sedis*, *Treponema*, *UCG_010*, *Gordonibacter*, *Candidatus Arthromitus* and *Marvinbryantia* were significantly enriched in the C + L + A group (Fig. 6E). The abundance of *Bacteroides*, *Lachnoclostridium*, *Romboutsia*, *Staphylococcus*, *Acetivibrio ethanolgignens group*, *Butyricicoccus*, *Bilophila*, *Eubacterium nodatum group*, *Chlamydia*, *Erysipelatoclostridiaceae*, *Streptococcus* and *Enterobacter* were significantly enriched in the C + L + A + P group (Fig. 6E). Combined with the above results, we found that *Akkermansia* and *Escherichia_Shigella* may be potential microorganisms for Polyphyllin B and FMT treatment in CSE and LPS-induced mice. However, the application of ABX may limit the effects of *Akkermansia* and *Escherichia_Shigella*, and change the composition of intestinal microbiota in C + L and C + L + P mice. In addition, compared with C + L mice, C + L + P and C + L + A + P treatment improved lung tissue injury and inhibited the levels of IFN-γ, IL-6 and TNF-α in BALF and lung tissue in C + L mice (Fig. 7A, B). Treatment with C + L + P and C + L + A + P also inhibited the deposition of Fe^3+^ and the expression of NCOA4 and p-STAT3, and promoted the GPX4 and ferritin protein expression in lung tissues of C + L mice (Fig. 7C-E). In conclusion, these results demonstrate that Polyphyllin B-derived FMT could inhibit ferritinophagy and ameliorate lung injury in CSE and LPS-induced mice.

### Polyphyllin B promoted Ferritinophagy in CSE and LPS-induced BEAS-2B cells

**Fig. 8:**
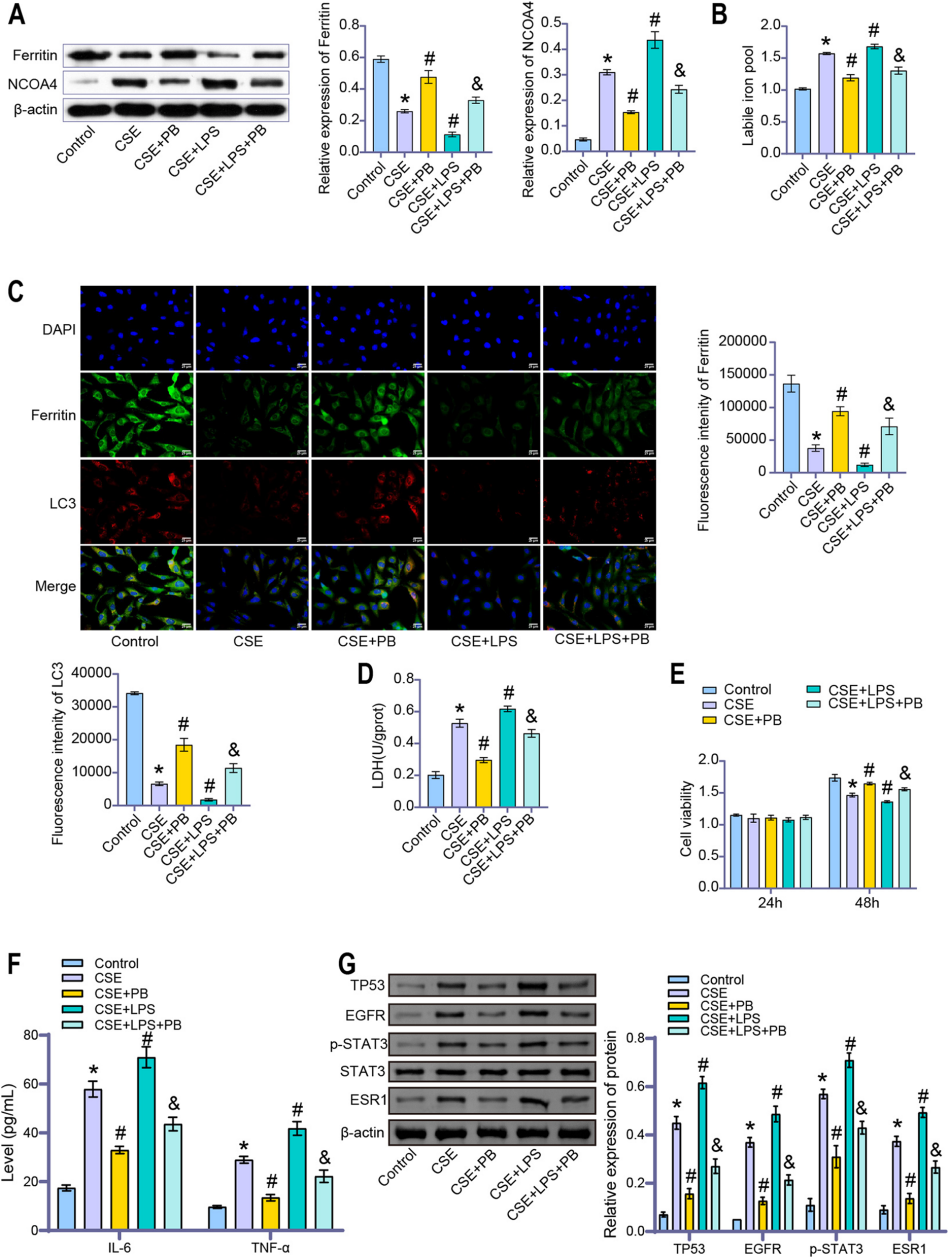
Polyphyllin B promoted CSE and LPS-induced Ferritinophagy in BEAS-2B cells. **A** The expressions of ferritin and NCOA4 protein were detected by western blot. Full-length blots/gels are presented in Supplementary Fig. 2E. **B** Calcein-AM method was used to detect the labile iron pool. **C** The expression of ferritin and LC3 was detected by immunofluorescence double staining. **D** LDH Assay kit was used to detect the change of LDH level. **E** CCK8 was applied to analyze cell viability. **F** The IL-6 and TNF-α levels were detected by ELISA. **G** The TP53, EGFR, STAT3 and ESR1 proteins were detected by western blot. Full-length blots/gels are presented in Supplementary Fig. 2F. *P < 0.05 vs Control, #*P* < 0.05 vs CSE, &*P* < 0.05 vs CSE + LPS. *N* = 3 replications/group

CSE induced the decrease of ferritin protein expression and the increase of NCOA4 protein expression in BEAS-2B cells, and LPS intervention aggravated the effect of CSE (Fig. 8A). Polyphyllin B treatment increased ferritin protein expression and decreased NCOA4 protein expression in BEAS-2B cells induced by CSE and CSE combined with LPS (Fig. 8A). The iron pool level of BEAS-2B cells was increased by CSE, and the effect of CSE was aggravated by LPS intervention (Fig. 8B). Polyphyllin B inhibited CSE and CSE combined with LPS induced iron pool levels in BEAS-2B cells (Fig. 8B). CSE induced the decrease of ferritin and LC3 protein expression, and LPS intervention aggravated the effect of CSE (Fig. 8C). Polyphyllin B promoted the expression of ferritin and LC3 proteins in BEAS-2B cells induced by CSE and CSE combined with LPS (Fig. 8C). Cytotoxicity and viability tests showed that CSE and CSE combined with LPS induced increased LDH levels and decreased cell viability in BEAS-2B cells (Fig. 8D, E). Polyphyllin B intervention down-regulates LDH levels and promotes cell proliferation (Fig. 8D, E). ELISA detection analysis showed that CSE and CSE combined with LPS induced an increase in IL-6 and TNF-α levels, but were suppressed by Polyphyllin B (Fig. 8F). We further validated the expression of genes ranked 5 in the interaction with COPD at the cellular level. Considering the host self-secretion type and function of ALB protein, we only validated the expression of TP53, EGFR, STAT3, and ESR1 proteins. The results showed that both CSE and CSE + LPS induced high expression of TP53, EGFR, STAT3, and ESR1 proteins in BEAS-2B cells, which were suppressed by the intervention of Polyphyllin B (Fig. 8G). These results demonstrated that Polyphyllin B promoted Ferritinophagy in BEAS-2B cells induced by CSE or CSE combined with LPS.

### Polyphyllin B promoted ferritinophagy in CSE + LPS-induced BEAS-2B cells by downregulating the STAT3/NCOA4 pathway

**Fig. 9:**
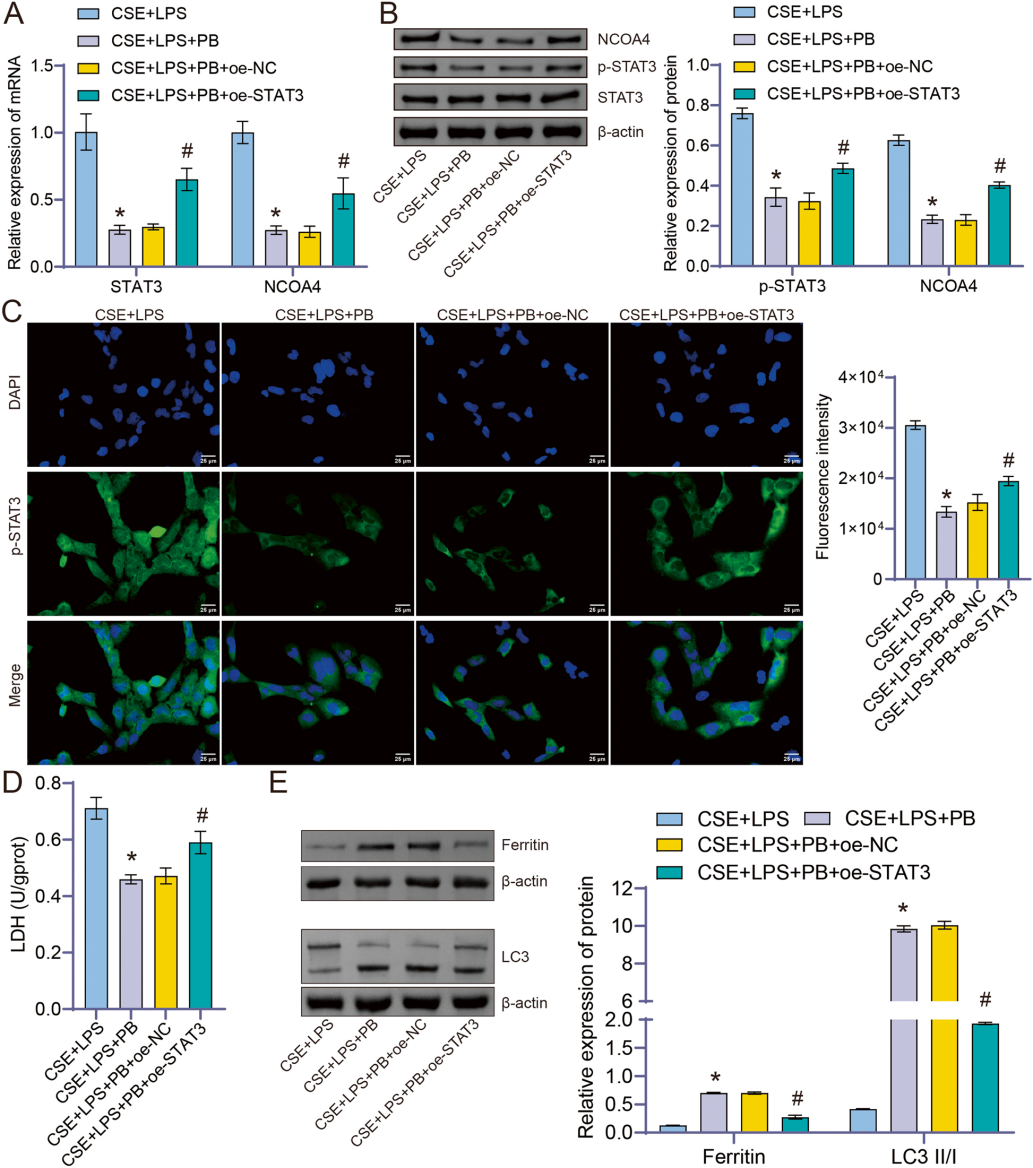
Polyphyllin B promoted ferritinophagy by down-regulating the STAT3/NCOA4 pathway. **A** PCR was used to detect the expression of STAT3 and NCOA4. **B** Western blot was performed to detect the expression of STAT3, p-STAT3, and NCOA4. Full-length blots/gels are presented in Supplementary Fig. 2G. **C** IF was used to detect the expression of p-STAT3. **D** LDH Assay kit was used to measure the changes in LDH levels. **E** Western blot was performed to detect the expression of ferritin and LC3. Full-length blots/gels are presented in Supplementary Fig. 2H. *P < 0.05 vs Model; #*P* < 0.05 vs Model + PB + oe-NC. *N* = 3 replications/group

Based on the results of molecular docking and animal experiments, we further explored the role of overexpressed STAT3 in the Polyphyllin B-treatment of CSE + LPS-induced BEAS-2B cell model. Polyphyllin B inhibited the expression of p-STAT3 and NCOA4 genes and proteins in CSE + LPS-induced BEAS-2B cells (Fig. 9A, B). Overexpressing STAT3 partially blocked the inhibitory effect of Polyphyllin B (Fig. 9A). Immunofluorescence detection confirmed that Polyphyllin B inhibited the phosphorylation of STAT3 in CSE + LPS-induced BEAS-2B cells, but it was reversed by oe-STAT3 (Fig. 9C). Moreover, overexpressing STAT3 weakened the inhibitory effect of Polyphyllin B on LDH levels in CSE + LPS-induced BEAS-2B cells (Fig. 9D, E). Overexpressing STAT3 also attenuated the promoting effect of Polyphyllin B on ferritin and LC3II/I expression in CSE + LPS-induced BEAS-2B cells (Fig. 9D, E). In conclusion, Polyphyllin B promotes ferritinophagy in CSE + LPS-induced BEAS-2B cells by downregulating the STAT3/NCOA4 pathway.

## Discussion

Ferroptosis is a regulated form of cell death that depends on iron and ROS, and is characterized by lipid peroxidation [31]. Ferroptosis played a key role in the pathogenesis of acute lung injury, COPD, pulmonary fibrosis, lung infections, and asthma [32]. In addition, ferroptosis was involved in the progression of sterile inflammation, such as chronic obstructive pulmonary disease, stroke, and ischemia–reperfusion injury caused by CS [33]. Previous studies have confirmed that ferroptosis was involved in CS-induced lung epithelial cell injury, inflammation and ferroptosis, which was reversed by curcumin [34]. Our study confirmed that Polyphyllin B up-regulated ferritin levels and improved lung injury induced by CSE or CSE combined with LPS in BEAS-2B cells and C + L mice. These results suggested that Polyphyllin B might be a new treatment for CSE + LPS-induced COPD by inhibiting ferroptosis.

Chronic inflammation in the lungs may result from a combination of genetic predisposition and environmental influences, including exposure to microorganisms, atmospheric particles, irritants, pollutants, allergens, and toxic molecules [35]. BEAS-2B cells and mouse lungs exposed to CSE/CS showed significant (*P* < 0.05) accumulation of polyubiquitin proteins and impaired autophagy markers in aggregates [36]. CS induced airway inflammation, airway remodeling and emphysema in experimental COPD mice [37]. CSE induced inflammation in 16HBE cells and Balb/c mice, and participates in oxidative stress and apoptosis through nuclear factor kappa B (NF-κB)/p65 pathway [38]. Our study found that CSE and LPS induced the increases of IFN-γ, IL-6 and TNF-α in BALF, the levels of LPS, TNF-α and IL-6 in peripheral blood, and the expression of TLR2, TLR4 and p65 protein in lung tissue, which were reversed by Polyphyllin B and FMT treatment. Polyphyllin B could effectively improve the pulmonary inflammation state of CSE and LPS-induced mice through gut microbiota.

COPD is primarily a tobacco smoke-induced disease, which is characterized by chronic low-grade systemic inflammation and lung aging (inflammation) associated with CS-mediated oxidative stress-induced steroid resistance [39]. CSE could induce oxidative stress in human bronchial epithelial 16HBE cells and mice, and reverse oxidative stress to prevent CS-induced airway remodeling in mice [40]. Resveratrol was known to have therapeutic effects in a rat model of COPD, which was associated with the inhibition of oxidative stress and inflammatory responses [41]. CS-related mitophagy induced excessive inflammation and drove programmed necrosis [42]. We found that administration of Polyphyllin B and FMT reduced the levels of ROS and nitrotyrosine in peripheral blood and lung tissue of CSE and LPS-induced mice, promoted the expression of ferritin and GPX4 in lung tissue, and inhibited the expression of NCOA4 and mitochondrial damage. These studies demonstrated that Polyphyllin B could reverse the oxidative stress, mitochondrial damage and ferritinophagy induced by CSE and LPS in mice through gut microbiota.

Polyphyllin B, also known as Formosanin C. Moreover, Formosanin C has chemotherapeutic potential against apoptosis-resistant HCC with a higher NCOA4 expression via ferritinophagy in liver cancer cells [43]. Animal studies have shown that Formosanin C also has immunomodulatory functions, reducing LPS-induced inflammation by inhibiting nuclear factor-κB in macrophages [44]. The STAT3-induced lysosomal membrane permeabilization-mediated autophagy can promote ferritinophagy [45]. Inhibiting or downregulating STAT3 can effectively reduce the levels of NCOA4, protecting H9C2 cells from ferritinophagy-mediated iron ferroptosis, while overexpression of STAT3 plasmid appears to increase the expression of NCOA4 and lead to classical iron ferroptosis events [46]. Our study proved that Polyphyllin B promoted ferritinophagy by down-regulating the STAT3/NCOA4 pathway. Accordingly, we speculate that promoting ferritinophagy appropriately may facilitate the rapid apoptosis and clearance of damaged lung cells induced by CSE + LPS, thus protecting lung cells from ferritinophagy-mediated iron ferroptosis. But the potential mechanism of Polyphyllin B needs to be further explored at the animal level.

In addition, the development of COPD was associated with an imbalance of 82 bacterial species, including *Escherichia_Shigella*, in plasma, BALF, and feces [47]. Xuanbai Chengqi decoction (XBCQ) significantly improved microbial homeostasis and inhibited inflammatory cell infiltration in COPD mice by accumulating probiotics *Gordonibacter* and *Akkermansia*, thereby alleviating lung inflammation [48]. Auricularia auricularia polysaccharide reduced the inflammatory injury of lung tissue caused by PM2.5 by restoring the taxonomic abundance of *Akkermansia* and down-regulating the IFN-γ, IL-4 and IL-8 contents [49]. Our study found that Polyphyllin B and FMT could down-regulate the levels of IFN-γ, TNF-α, and IL-6 and restore the abundance of *Akkermansia* and *Escherichia_Shigella* to improve the gut microbiota disorder and lung injury in CSE and LPS-induced mice. However, the application of ABX might limit the effects of the *Akkermansia* and *Escherichia_Shigella*, and reconstruct the gut microbiota composition of C + L and C + L + P mice.

About 20% of smokers develop COPD due to irreversible damage to the airway epithelium and persistent inflammation caused by smoking [50]. Currently, plant chemicals such as polyphenols and flavonoids have been proven to have medicinal effects on COPD [51]. Natural compounds, including medicinal plants and their derivatives, may be used to treat toxic substance-induced lung diseases by improving various characteristics of lung injury, but further research is still needed before clinical practice [52]. Clinical studies have shown that the combination treatment of curcumin and piperine has good effects on systemic oxidative stress, clinical symptoms, and health-related quality of life (HRQoL) in the treatment of chronic pulmonary complications, which can compensate for the low oral bioavailability of curcumin [53]. Additionally, co-administration with piperine, incorporation into micelles, micro/nanoemulsions, nanoparticles, liposomes, solid dispersions, spray drying, and formation of non-covalent complexes with saponins can improve the oral bioavailability of curcumin [54]. These ideas are worth considering and applying to the improvement of cigarette smoke + LPS-induced mouse lung injury using Polyphyllin B. Our preliminary research has confirmed the therapeutic effects of Polyphyllin B on inflammation, oxidative stress, and lung injury in cigarette smoke + LPS-induced mice, but its clinical application still needs further investigation.

## Conclusion

Therefore, our study proved that Polyphyllin B could improve lung tissue injury in CSE and LPS-induced mice by restoring gut microbiota disorder and inhibiting STAT3/NCOA4 pathway. This may be an effective strategy to improve cigarette smoke-related COPD.

### Supplementary Information

**Supplementary Material 1**.

## Data Availability

All data included in this study are available upon request by contact with the corresponding author.
