# Supplementary material for: Polyphyllin B inhibited STAT3/NCOA4 pathway and restored gut microbiota to ameliorate lung tissue injury in cigarette smoke-induced mice
Source: BMC Biotechnol. 2024 Mar 8;24:13. doi: 10.1186/s12896-024-00837-6 (PMC10921762; doi:10.1186/s12896-024-00837-6)
Supplement: Supplementary file 1 — Supplementary Material 1. [file 12896_2024_837_MOESM1_ESM.docx]

**
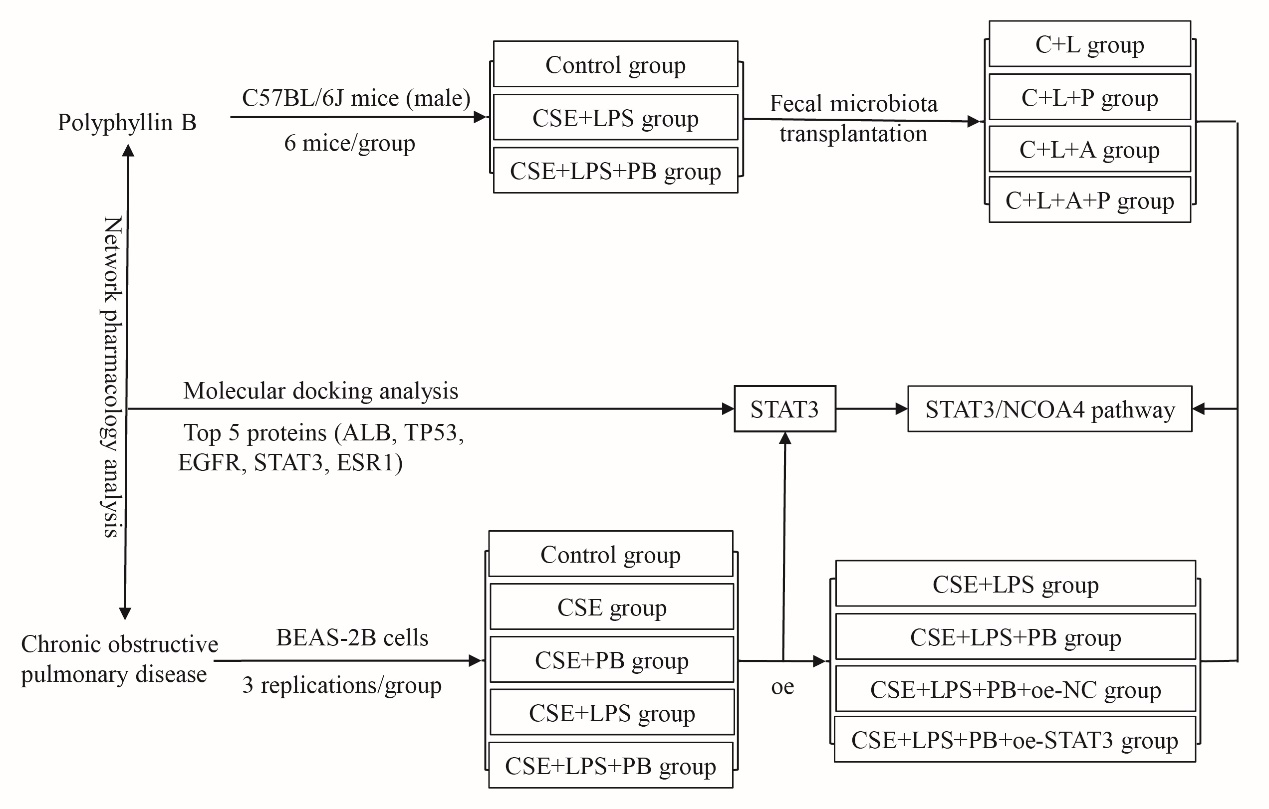
**

**Supplementary Figure 1. The flowchart of the study.**

**
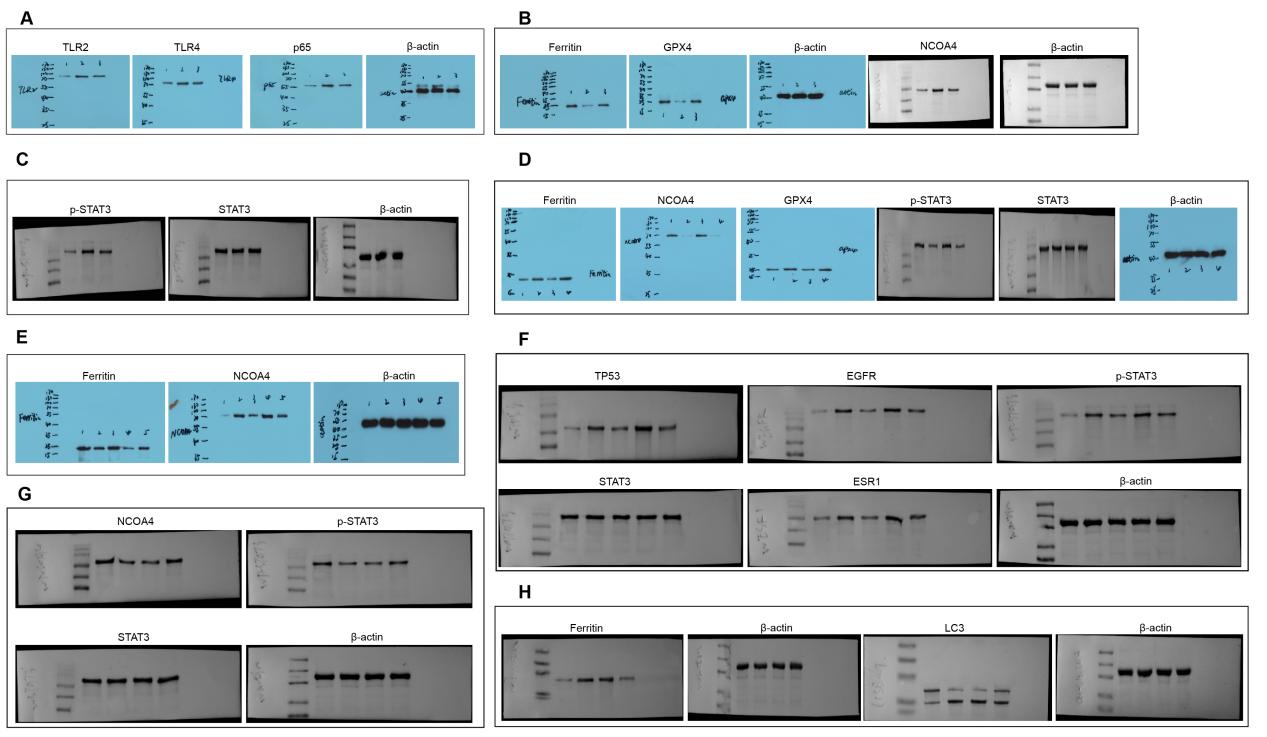
**

**Supplementary Figure 2. Full-length blots of each western blot.** (A) Full-length blots of Figure 2F. (B) Full-length blots of Figure 3B. (C) Full-length blots of Figure 3C. (D) Full-length blots of Figure 7E. (E) Full-length blots of Figure 8A. (F) Full-length blots of Figure 8G. (G) Full-length blots of Figure 9B. (H) Full-length blots of Figure 9E.
